# Deciphering transcriptional regulations coordinating the response to environmental changes

**DOI:** 10.1186/s12859-016-0885-0

**Published:** 2016-01-16

**Authors:** Vicente Acuña, Andrés Aravena, Carito Guziolowski, Damien Eveillard, Anne Siegel, Alejandro Maass

**Affiliations:** Center for Mathematical Modeling (UMI-CNRS 2807), Universidad de Chile, Santiago, Chile; Center for Genome Regulation, Universidad de Chile, Santiago, Chile; Department of Mathematical Engineering, Universidad de Chile, Santiago, Chile; Department of Molecular Biology and Genetics, Istanbul University, Istanbul, Turkey; IRCCyN (UMR CNRS 6597), École Centrale de Nantes, Nantes, France; LINA (UMR CNRS 6241), Université de Nantes, École des Mines de Nantes, Nantes, France; IRISA Project Dyliss (UMR CNRS 6074), Université de Rennes 1, Rennes, France

**Keywords:** Transcriptional regulatory network, Co-expression, Combinatorial graphs

## Abstract

**Background:**

Gene co-expression evidenced as a response to environmental changes has shown that transcriptional activity is coordinated, which pinpoints the role of transcriptional regulatory networks (TRNs). Nevertheless, the prediction of TRNs based on the affinity of transcription factors (TFs) with binding sites (BSs) generally produces an over-estimation of the observable TF/BS relations within the network and therefore many of the predicted relations are spurious.

**Results:**

We present Lombarde, a bioinformatics method that extracts from a TRN determined from a set of predicted TF/BS affinities a subnetwork explaining a given set of observed co-expressions by choosing the TFs and BSs most likely to be involved in the co-regulation. Lombarde solves an optimization problem which selects confident paths within a given TRN that join a putative common regulator with two co-expressed genes via regulatory cascades. To evaluate the method, we used public data of *Escherichia coli* to produce a regulatory network that explained almost all observed co-expressions while using only 19 % of the input TF/BS affinities but including about 66 % of the independent experimentally validated regulations in the input data. When all known validated TF/BS affinities were integrated into the input data the precision of Lombarde increased significantly. The topological characteristics of the subnetwork that was obtained were similar to the characteristics described for known validated TRNs.

**Conclusions:**

Lombarde provides a useful modeling scheme for deciphering the regulatory mechanisms that underlie the phenotypic responses of an organism to environmental challenges. The method can become a reliable tool for further research on genome-scale transcriptional regulation studies.

**Electronic supplementary material:**

The online version of this article (doi:10.1186/s12859-016-0885-0) contains supplementary material, which is available to authorized users.

## Background

Deciphering the mechanisms that explain the coordinated change in gene expression of an organism as a response to changes in the environment, is one of the fundamental challenges in systems biology. Moreover, high-throughput expression data have provided evidence that these mechanisms act in intriguing ways to coordinate gene expression, emphasizing the complexity of the regulation as part of the acclimation process.

In general, classic methods of *in silico* reconstruction of transcriptional regulation processes consider expression profiles and genomic sequences separately. The most commonly used strategies for identifying co-expressed genes consider linear correlation [1] or mutual information methods like ARACNe [2], CLR [3], and MRNET [4]. Some of these methods have been successful in identifying regulatory interactions in synthetic networks and in model organisms [5]. Nonetheless, the interdependence of the expression profiles of two genes does not necessarily mean that there is a physical interaction. Also, the computed correlations are not oriented and thus cannot be interpreted causally. Even so, they convey information about the transcriptional mechanisms.

On the other hand, different *in silico* approaches have been developed for the study of genomic sequences. These approaches attempt to model the physical interactions that form a putative transcriptional regulatory network (TRN) and they rely on the identification of pairs of genes where the first gene codes for a transcription factor (TF) that potentially binds in the promoter region of the second gene [6, 7]. Genes coding for TFs are typically obtained by homology between the genome sequence and a database of TFs (such as RegulonDB [8] or Prodoric [9]). In these databases, each TF has an associated *position weighted matrix* (PWM), which estimates the affinity between the TF and a potential binding site (BS) in a promoter region. The low specificity of current methods for identifying transcriptional regulations means that the number of TF/BS affinities found is usually huge, while, in practice, only a few of them correspond to observed regulatory interactions. Nevertheless, even if many of the predicted interactions have a low probability of occurrence, or are never observed, it is reasonable to assume that the TRN reconstructed from them contains most of the physical interactions that occur for a given process and therefore the network should be able to explain co-expression of genes. Moreover, the *p*-value of the affinity computed for a given TF/BS interaction provides information that can be interpreted as a *likelihood* of the occurrence of this interaction.

Under this assumption, the problem we address here is: given a putative TRN constructed from TF/BS affinities and given a set of co-expressed gene pairs, how can the most probable set of interactions that the organism uses to coordinate the gene expression changes be determined. In other words, we want to find a *simple and confident* subnetwork of a putative TRN that is able to *explain* a given set of co-expressions.

**Fig. 1:**
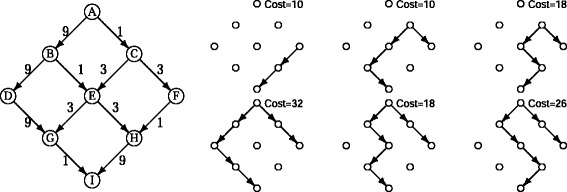
Simple representation of the notion of explanation. *Left:* A TRN representing 12 TF/BS affinity interactions between 9 genes. Costs 1, 3, and 9 are associated according to their *p*-values (*k*=3, *r*=3). *Right:* Six possible explanations for a co-expressed pair (F,I); two of them are optimal explanations (cost =10)

To define what we mean by a subnetwork *explaining* a pair of co-expressed genes, we consider all the topological configurations within the network that allow for a coordinate change. The simplest case is when a pair of co-expressed genes also corresponds to a TF/BS interaction in the putative TRN. Clearly a direct interaction, represented in the TRN as a single arc between the genes, is a possible explanation for the co-expression, indicating that one gene is regulating the expression of the other gene. Another possibility is that the co-expression of the two genes is correlated not by a single regulatory interaction but by a chain of TF/BS interactions, *i.e.* a regulatory cascade. Such a cascade is represented in the TRN as a directed path from one gene to the other. Thus, a path connecting two correlated genes is also a possible explanation for the co-expression. Finally, the correlation may be the result of a third gene that simultaneously regulates two co-expressed genes. In such a situation, the two correlated genes will be at the ends of two regulatory cascades, both of which start with this common regulator gene (see right side of Fig. 1). We define any of these configurations as an *explanation* for the correlation.

To identify a *simple and confident* subnetwork explaining a set of co-regulations, we propose LOMBARDE, an optimization strategy that extracts from a putative TRN the most simple and reliable TF/BS interactions that explain a given set of co-regulated genes. The LOMBARDE method also accepts as input an additional independent list of experimentally validated transcriptional regulations.

The precision of LOMBARDE clearly depends on our assumption that the initial putative TRN includes most of the observed TF/BS interactions. When this assumption is satisfied, at least one explanation for each real co-expression should be assured. Interestingly, we found that a putative TRN constructed for *Escherichiacoli* using a classical bioinformatic pipeline to produce TF/BS affinity pairs explained 91.1 % of nearly 60,000 observed co-expressions (see Results and discussion for details).

When LOMBARDE was applied to this putative *E.coli* TRN and the set of observed co-expressions it produced a subnetwork that conserved only 19.2 % of the initial interaction arcs, while still explaining 91.1 % of the co-expressions. LOMBARDE has a strong bias towards preserving experimentally validated regulations. It preserved over 66 % of a set of independent experimentally validated arcs in the putative *E.coli* TRN and kept only 18.4 % of non-validated interactions. Moreover, when LOMBARDE was applied to the same putative TRN extended by adding all independent experimentally validated arcs, the resulting subnetwork retained 92 % of the validated arcs and included only 11.3 % of the other putative regulations. In addition, the subnetworks produced by LOMBARDE showed credible topological characteristics and recovered most of the global regulators described in the *E. coli* literature. The regulators were also ranked correctly in relation to their role in the network. We concluded that the modeling scheme proposed in LOMBARDE is a reliable strategy for deciphering the transcriptional regulatory interactions that can explain the co-expressions observed under environmental changes.

## Materials and methods

Given a putative TRN computed from TF/BS affinities and a set of co-expressed gene pairs, the main idea of LOMBARDE is to extract from this network a simple and confident subnetwork that contains an explanation for each co-expression in the given set. Here we present a description of the proposed model and the methods used. We also compare the optimization strategy defined to model simple and confident subnetworks with alternative optimization strategies.

### Input of LOMBARDE

If $\mathbb {G}$ is the set of genes in a studied organism, then LOMBARDE requires the following inputs:

1. *Co-expressed pairs:* A set $\mathcal {C}\subseteq \mathbb {G}\times \mathbb {G}$ of pairs of co-expressed genes, selected based on the values of their correlation or mutual information. An example of such a set would be the results of ARACNe [2], MRNET [4], or other mutual information based methods evaluated using expression profiles obtained under different environmental conditions.
2. *Affinity pairs:* A set $\mathcal {A}\subseteq \mathbb {G}\times \mathbb {G}$ of gene pairs obtained based on TF/BS sequence affinity and the associated *p*-values. Specifically, a pair of genes (*A*,*B*) is in $\mathcal {A}$ if gene *A* codes for a TF that has high affinity with a BS in the promoter region of gene *B*. For instance, $\mathcal {A}$ could be the result of matches in the Prodoric database [9]. We assume that pairs with high *p*-values would already have been discarded from $\mathcal {A}$.
3. *Validated pairs:* Optionally, a set $\mathcal {V}\subseteq \mathbb {G}\times \mathbb {G}$ of gene pairs that correspond to independent experimentally validated regulations, if available.

LOMBARDE was initially intended to be applied in Prokarya where all the genes of a given operon are transcribed typically in a single polycistronic mRNA molecule; therefore, we assumed that the expression of a gene implies the expression of the operon to which it belongs. Given the specific operon structure of the studied organism, *E.coli*, we can consider $\mathbb {G}$ as the set of operons and $\mathcal {C}$, $\mathcal {A}$ and $\mathcal {V}$ as sets of pairs of operons, which simplifies the analysis and reduces the running time of the method. This simplification is purely operational and can be applied at the user’s discretion.

### Defining the *a priori* graph $\mathcal {G}$ and explanations

Initially, LOMBARDE defines the *a priori graph*$\mathcal {G}$ as a directed graph where nodes correspond to genes $\mathbb {G}$ and directed arcs correspond to pairs of affinities in $\mathcal {A}$ and pairs of known regulations in $\mathcal {V}$. That is, $\mathcal {G}=(\mathbb {G},\mathcal {A}\cup \mathcal {V})$. Thus, there will be a directed arc from gene *A* to gene *B* if there is some *a priori* evidence (experimental or theoretical, weak or strong) that *A* directly regulates *B*. If no validated regulations are available, then $\mathcal {G}=(\mathbb {G},\mathcal {A})$. It is important to note that a regulatory cascade, *i.e.* a sequence of regulatory relations between genes in $\mathcal {G}$, should appear in this graph as a directed path (see the right side of Fig. 1), although clearly not every path will represent a real regulatory cascade. The final objective is to highlight paths that most likely correspond to real regulatory cascades controlling the co-expressed data.

Under this representation of a TRN, the observed co-regulation of two genes in $\mathcal {C}$ can be explained by considering two cases. One, is the existence of a directed path from one gene to the other, meaning that the first gene is regulating the last gene through a regulatory cascade (direct regulation is considered as a regulatory cascade of size one). Two, is considering that none of the genes regulate the other, rather both are co-regulated by a third gene. Such a situation is represented in the *a priori* graph by two paths from a common regulator to each of the co-regulated gene (see right side of Fig. 1).

#### **Definition1**.

Given a pair $(A,B) \in \mathcal {C}$ of co-expressed genes, an *explanation for (A,B)* in $\mathcal {G}$ is a set of arcs $\mathcal {E}$ that satisfy any of the following conditions:

- $\mathcal {E}$ is a directed path from *A* to *B*;
- $\mathcal {E}$ is a directed path from *B* to *A*;
- $\mathcal {E}$ is the union of two divergent directed paths starting from a gene *C* and arriving, respectively, at *A* and *B*, which have only vertex *C* in common.

#### **Definition2**.

We say that a subgraph $\mathcal {G}^{\prime }\subseteq \mathcal {G}$ explains $\mathcal {C}$ if, for every pair $(A,B)\in \mathcal {C}$, the subgraph $\mathcal {G}^{\prime }$ contains an explanation for (*A*,*B*).

Ideally, every pair (*A*,*B*) in $\mathcal {C}$ should have at least one explanation in $\mathcal {G}$. If this is not the case, it indicates that, under the modeling hypothesis, *A* and *B* are not really co-regulated or that the methods used to compute the set $\mathcal {A}$ did not capture all the transcriptional mechanisms involved in the co-regulation of *A* and *B*. These unexplained pairs correspond to missing or inaccurate input data beyond the scope of the method and therefore are removed from $\mathcal {C}$. After their removal we can assume that the *a priori* graph $\mathcal {G}$ explains $\mathcal {C}$. However, as discussed earlier, many arcs in $\mathcal {G}$ represent TF/BS relations that have a low probability of occurrence or that have never been observed. Our objective, therefore, is to find a *simple and confident* subgraph $\mathcal {G}'\subseteq \mathcal {G}$ that explains every pair in $\mathcal {C}$.

### Cost definition

We considered two ways of defining simple and confident subgraphs of $\mathcal {G}$ that explain $\mathcal {C}$: (i) foster explanations that use a small number of arcs and (2) foster explanations that have arcs of high affinity (*i.e.* low *p*-value). A way to consider both criteria simultaneously is to define costs on the arcs in such a way that the most likely TF/BS affinities have the lowest costs.

Instead of defining the cost of an arc in $\mathcal {G}$ as a continuous function of the *p*-value of the associated TF/BS, we implemented a qualitative approach by defining *levels of likelihood*. Indeed, because small variations in *p*-values have no real biological significance, we considered all arcs with similar *p*-values as equally likely. This approach increased the robustness of the method and prevented solutions that were slightly but not significantly different to the best ones from being discarded.

We defined the costs of the arcs in $\mathcal {G}$ using the following procedure where $k \in {\mathbb N}$ and *r*∈(0,*∞*) are parameters. We defined *k* levels of likelihood, from level *i*=0 (highest likelihood) to level *i*=*k*−1 (lowest likelihood), in such a way that every level contains the same number of arcs (*i.e.* in equal-frequency bins). Arcs in $\mathcal {V}$ are assigned to the highest likelihood level (valued *i*=0), because they correspond to already validated regulations. Finally, the cost of an arc in level *i*∈{0,…,*k*−1} is set as *r*^*i*^. In this way *r* represents the incremental cost between consecutive levels; that is, an arc in level *i* has a cost that is equal to *r* arcs in level *i*−1. We analyzed the use of different values for the parameters *k* and *r* on a real data sets (see the ‘Analysis of cost parameters’ subsection for details).

### Optimal explanations and subgraphs

Having defined the cost of arcs, it is natural to define the cost of a subgraph as the sum of the costs of the arcs it contains. With this, we defined an *optimalexplanation*:

#### **Definition3**.

We define an explanation $\mathcal {E}$ for the pair (*A*,*B*) in $\mathcal {C}$ as *optimal* if it has the minimum cost among all the explanations for the pair.

**Fig. 2:**
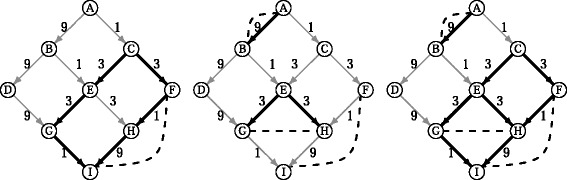
LOMBARDE output for the co-expressions (F,I), (A,B), and (G,H). *Left:* LOMBARDE computes the two optimal explanations for (F,I). *Center:* LOMBARDE continues computing the optimal explanation for (A,B) and for (G,H). *Right:* The output $\mathcal {G}_{L}$ is the union of all optimal explanations

Note that a pair (*A*,*B*) in $\mathcal {C}$ can have more than one optimal explanation (see Fig. 2), especially after the cost categorization where all arcs with similar *p*-values are considered as equally likely. Thus we define an *optimal subgraph*:

#### **Definition4**.

We define a subgraph $\mathcal {G}^{\prime }\subseteq \mathcal {G}$as an optimal subgraph explaining $\mathcal {C}$ if $\mathcal {G}^{\prime }$ is the union of $|\mathcal {C}|$ (cardinality of $\mathcal {C}$) optimal explanations, one for each pair of genes in $\mathcal {C}$. That is,

$$\mathcal{G}^{\prime}=\bigcup_{(A,B) \in \mathcal{C}} \mathcal{E}_{(A,B)}, $$

where $\mathcal {E}_{(A,B)}$ is an optimal explanation for (*A*,*B*).

There could be a huge number of optimal subgraphs explaining $\mathcal {C}$, because several optimal explanations for each pair (*A*,*B*) in $\mathcal {C}$ could exist. For example, if $\mathcal {C}$ contained 20 gene pairs, each one having two optimal explanations, then the number of optimal subgraphs explaining $\mathcal {C}$ could reach a million (when each possible union of optimal explanations produced a different subgraph).

Instead of enumerating all optimal subgraphs explaining $\mathcal {C}$, which could be computationally infeasible, we computed a subgraph $\mathcal {G}_{L}$ defined as the union of all optimal subgraphs explaining $\mathcal {C}$. It is clearly not necessary to compute every optimal subgraph, but rather to compute for every (*A*,*B*) pair in $\mathcal {C}$ only the set of all optimal explanations for (*A*,*B*). Thus, the graph $\mathcal {G}_{L}$ is obtained as,

$${}\mathcal{G}_{L} = \bigcup_{(A,B)\in\mathcal{C}} \{\mathcal{E} \ | \ \mathcal{E}\ \text{is an optimal explanation of}\, (A,B)\, \text{in}\, \mathcal{G}\}. $$

This graph is the output of LOMBARDE (see Fig. 2 for an example).

### Analysis of alternative optimization problems

We proposed $\mathcal {G}_{L}$ as a simple and confident subgraph of $\mathcal {G}$ to explain the co-expressions provided in $\mathcal {C}$ by including all the optimal explanations for each pair in $\mathcal {C}$. This can be seen as a *local* optimization problem because the cost of explaining every pair in $\mathcal {C}$ independently is minimized. The main reason for choosing this local strategy was that other natural alternatives that consider solving *global* optimization problems are computationally infeasible [10].

**Fig. 3:**
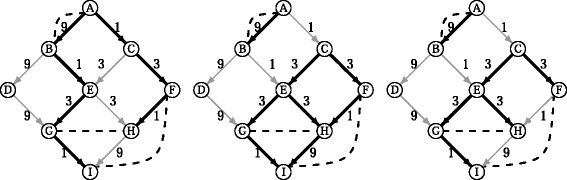
Comparison between LOMBARDE output and an alternative minimum global cost explanations. *Left:* Graph with minimal cost that explains all pairs of co-expressions. Note that the explanation proposed for (G,H) has cost 18, which is much higher than the optimal explanation of cost 6. *Center:* LOMBARDE output all optimal explanations for each pair. *Right:* Minimum cost subgraph containing an optimal explanation for each pair. This graph is always contained in the LOMBARDE output

For instance, consider the problem of computing the *subgraph of minimum cost that explains*$\mathcal {C}$. This criterion can give a different result than $\mathcal {G}_{L}$ (see Fig. 3 for an example). However, this approach is not robust because adding a new co-expression pair could completely change the global solution. Moreover, the best explanation that it provides for a given pair could have a very high cost compared with an optimal explanation. As well as having these undesirable properties, this problem is hard to solve. Indeed, it has been shown to be an NP-hard problem [10] by a reduction from the *Steiner Weighted Directed Tree* problem. This means that the problem can be solved exactly and in a reasonable time only for very small instances.

Another alternative optimization problem, which can be considered as a mixture between local and global optimization, is to compute a minimum cost subgraph that contains an optimal explanation for each pair in $\mathcal {C}$; that is, the optimal subgraph explaining $\mathcal {C}$ with minimum global cost. Although this approach may seem more interesting than the previous alternative, it has some disadvantages. One disadvantage is the possible multiplicity of solutions because again there could be a large number of optimal subgraphs that have the same minimum global cost. An even worse disadvantage is that it has been proved by a reduction from the *Minimum Hitting Set* problem that finding just one solution is NP-hard [10] (although in practice this problem can be solved for larger instances). Besides the disadvantages, the results produced by this alternative optimization are not more interesting in practice than those given by LOMBARDE because they always correspond to subsets of $\mathcal {G}_{L}$. Thus, LOMBARDE gives not only the global optimal solution of the optimization problem but also provides alternative optimal explanations for each pair. Moreover, the solution given by LOMBARDE is always unique and computationally feasible.

## Results and discussion

A large number of TF/BS associations have been validated experimentally for *E.coli*; therefore, we used public data for this bacteria to evaluate our model. The genome sequence and gene annotation of *E.coli* K12 [GenBank:NC_000913] were downloaded from the NCBI ftp site [11] (http://www.ncbi.nlm.nih.gov/nuccore/NC_000913.3). Differential expression profiles for 907 conditions were obtained from Ecoli_v4_Build_6_chips907probes4297.tab the Many Microbes Microarray Database [12] http://m3d.mssm.edu/norm/E_coli_v4_Build_6.tar.gz.

### Input data

Here, we describe briefly the sources and methods used to generate input data for LOMBARDE. As mentioned above, for bacteria we condense the information into operons to simplify computation. It is important to note that the methods we used to produce the input for LOMBARDE were merely a choice. Because data production was not our main concern, as far as possible, we used simple pipelines that are currently used by the community to build the putative TRNs.

■ $\mathcal {C}$: **Pairs of co-expressed operons:** A set of 61,506 pairs of co-expressed operons was generated by analyzing the *E.coli* differential expression profiles as follows. A matrix with the mutual information between all pairs of the 4297 *E. coli* genes was computed using a parametric Gaussian density estimator with the minet [13] library of the R statistical package [14]. The matrix had over 18 million values, although most of them were either insignificant or redundant and were discarded using the MRNET [4] strategy. From the remaining pairs of genes we considered only the 100,000 that had the highest mutual information, *i.e.* about 5 % of the total pairs. This number was chosen to support the inclusion of the main co-expressions. Finally, two operons were considered to be co-expressed if each one contained a gene from a pair of co-expressed genes. After discarding redundant and trivial cases, we obtained a set of 61,506 pairs of co-expressed operons, involving 2492 different operons.
■ $\mathcal {A}$: **Affinity network and its p-values**: A set of 25,604 pairs of operons with high TF/BS affinity was produced as follows. Genes coding for TFs were identified by BLAST homology searches (E-value cutoff of 10^−10^) between the gene product and a known instance of the TF in the Prodoric database [9] (http://www.prodoric.de). Then for each TF, a BS was considered each time a putative binding site in the upstream region (up to 300 bp) of a gene was located by MEME/FIMO [15] with a *p*-value smaller than 10^−5^. A pair of operons was reported if the first operon contained a gene coding for a TF with a putative BS in the upstream region of the second operon. The *p*-value for a pair was defined as the *p*-value of the affinity represented. (The minimum *p*-value was used if more than one TF in the first operon had affinity with the promoter region of the second operon.). The set of 25,604 pairs involved 2390 different operons.
■ $\mathcal {V}$: **Validated network:** A set of 1652 pairs of operons that represent independent experimentally validated transcriptional regulations was generated. Each pair of operons was reported if the first operon contained a gene coding for a TF that regulated the expression of the second operon based on the compilations made by Salgado et al. [8, 16] available at http://regulondb.ccg.unam.mx/. This set of 1652 pairs contained a total of 823 different operons.

We used this data to build the *a priori* graph that is used by LOMBARDE. Then we simulated two possible application scenarios.

■ ($\mathcal {G}_{\mathcal {A}}$) **Ab initio**: This scenario simulates a case when no validated regulations are available; therefore, only co-expressions $\mathcal {C}$ and affinities $\mathcal {A}$ are used as input. The *a priori* graph $\mathcal {G}_{\mathcal {A}}$ generated from this input contained 25,604 arcs corresponding to the affinities in $\mathcal {A}$. Although the set of independently validated regulations $\mathcal {V}$ is discarded in the input of Lombarde, this set of independent information is used to evaluate the bias of the method to include confirmed regulations, because 444 regulations in $\mathcal {A}$ were also in $\mathcal {V}$.
■ ($\mathcal {G}_{\mathcal {AV}}$) **Extended**: This scenario considers all the data $\mathcal {C}, \mathcal {A}$, and $\mathcal {V}$ as input and uses Lombarde in the usual way. The *a priori* graph $\mathcal {G}_{\mathcal {AV}}$ generated from this input contained 26,812 arcs, corresponding to the union $\mathcal {A}\cup \mathcal {V}$ (444 pairs are in the intersection, *i.e.* TF/BS affinities that were also experimentally validated). In this case all arcs in $\mathcal {V}$ are assigned cost 1, corresponding to the highest likelihood.

To evaluate the results of LOMBARDE compared with the already known TRN, we defined the *validated regulatory network* as the graph $\mathcal {G}_{\mathcal {V}}$ that had only the arcs in $\mathcal {V}$.

### Explanatory potential of *E.colia priori* graph

If we consider the validated regulatory network $\mathcal {G}_{\mathcal {V}}$ (composed with just the validated arcs in $\mathcal {V}$), only 3990 co-expressions (6.5 % of $\mathcal {C}$) were explained. The main reason for this low value is that the set of co-expressed pairs $\mathcal {C}$ involved 2492 different operons, while the network of validated regulations contained only 823 different operons. Thus, most of the operons in $\mathcal {C}$ were not contained in the network of validated regulations. Interestingly, among the 3990 explained co-expressions, only 83 were explained by a single validated arc, while the remainder were explained only through regulatory cascades. This result is consistent with the results in Sun et al. [17] and shows that the reconstruction of a TRN using expression data alone seems to be infeasible, and confirms the role of regulatory cascades.

On the other hand, when the *ab initio* scenarios (computed affinities in $\mathcal {A}$) were considered we found that the *a priori* graph $\mathcal {G}_{\mathcal {A}}$ explained 56,044 of the pairs of co-expressed operons (*i.e.* 91.1 % of $\mathcal {C}$). This number rose to 56,789 (92.3 % of $\mathcal {C}$) in the extended case when validated arcs were included in the *a priori* graph $\mathcal {G}_{\mathcal {AV}}$. This result reveals the explanatory potential of the *a priori* graphs $\mathcal {G}_{\mathcal {A}}$ and $\mathcal {G}_{\mathcal {AV}}$.

Nevertheless, the huge number of affinities (more than 15 times the number of known regulations) is consistent with the evidence that many of the predicted TF/BS affinities are spurious and they are not part of the real regulatory processes that coordinate the gene co-expressions given as input, which is obtained from a particular set of experiments. The modeling principle of LOMBARDE is that we can choose the most confident subnetwork which explains the studied data.

### LOMBARDE results are biased towards validated interactions

Considering the *ab initio* scenario, LOMBARDE was first applied to $\mathcal {G}_{\mathcal {A}}$ and the set $\mathcal {C}$ of observed co-expressions for *E.coli*. After setting the cost parameters *k*=9 and *r*=10 (an analysis of this choice is presented below), LOMBARDE produced a subnetwork with only 19.2 % of the initial arcs (4922 of 25,604), which still explained 91.1 % of the co-expressions. Interestingly, LOMBARDE showed a strong bias towards preserving independent experimentally validated regulations in $\mathcal {V}$ (see Table 1). Indeed, LOMBARDE preserved 66.4 % of the validated arcs in $\mathcal {G}_{\mathcal {A}}$ and kept only 18.4 % of the non-validated interactions. A hypergeometric test confirmed this bias, with an enrichment *p*-value under 10^−105^. For the extended scenario, this bias was even stronger when LOMBARDE was applied to the extended graph $\mathcal {G}_{\mathcal {AV}}$ (*i.e.*, adding all validated regulations). The resulting subnetwork kept 92 % of the validated arcs (1520 of 1652) and included only 11.3 % of the non-validated putative regulations (2854 of 25,604). For a future work, it would be interesting to explore whether the 2,854 putative regulations contain real regulatory relations in *E.coli* which have not been experimentally validated yet.

**Table 1:** Characteristics of the *a priori* graphs and LOMBARDE output networks

Network	Explained		No. of	No. of	No. of
	co-expressions		vertices	arcs	arcs in $\mathcal {V}$
TRN $\mathcal {G}_{\mathcal {V}}$ built from $\mathcal {V}$	3,990	(6.5 %)	823	1,652	1,652
*E. coli* *ab initio* $\mathcal {G}_{\mathcal {A}}$	56,044	(91.1 %)	2,390	25,604	444
Lombarde output for $\mathcal {G}_{\mathcal {A}}$	56,044	(91.1 %)	2,336	4,922	295
*E. coli* extended$\mathcal {G}_{\mathcal {AV}}$	56,789	(92.3 %)	2,434	26,812	1,652
Lombarde output for $\mathcal {G}_{\mathcal {AV}}$	56,789	(92.3 %)	2,370	4,374	1,520

The a priori graphs explained most of the co-expressions. The LOMBARDE results kept most of the vertices, significantly reduced the number of arcs, and kept most of the validated arcs

**Fig. 4:**
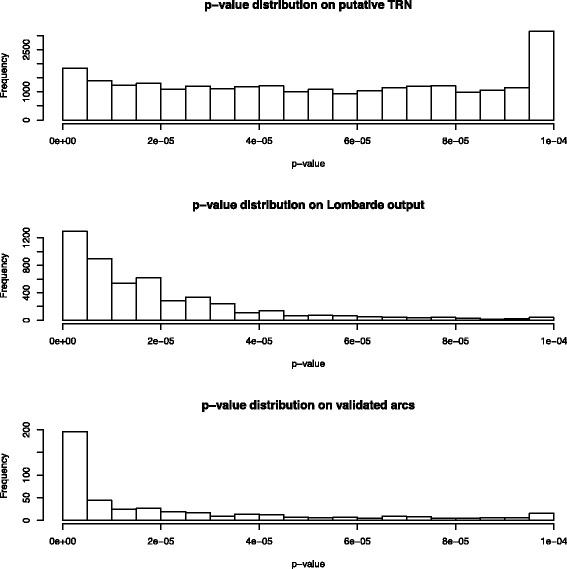
Distribution of the *p*-values of the TF/BS affinities in the *a priori* graph $\mathcal {G}_{\!\mathcal {A}}$, the output of LOMBARDE when applied to $\mathcal {G}_{\mathcal {A}}$ and the TRN $\mathcal {G}_{\mathcal {V}}$ constructed from $\mathcal {V}$ are shown

It should be noted that while LOMBARDE preferentially chooses arcs with low *p*-values, it also includes arcs with high *p*-values when they are required to explain a co-expression (see Fig. 4). Validated arcs are also biased towards lower *p*-values, although some do have high values. Thus methods based on only a *p*-value threshold will not recover all validated arcs and may not produce the largest networks.

We expected that LOMBARDE would recover many non-validated arcs because sets of validated regulations represent only the current knowledge, which may correspond to a very small portion of all the transcriptional regulations in an organism.

### Degree distribution of LOMBARDE output are similar to observed TRNs

**Fig. 5:**
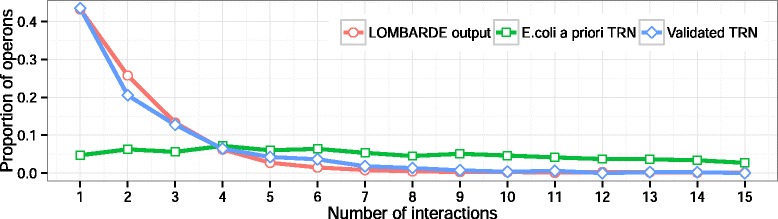
Degree distributions of the networks. The *a priori* graph $\mathcal {G}_{\mathcal {A}}$ (green), output of LOMBARDE when applied to $\mathcal {G}_{\mathcal {A}}$ (red), and the TRN $\mathcal {G}_{\mathcal {V}}$ constructed from $\mathcal {V}$ (blue) are shown

Some characteristics that were measured in the LOMBARDE results suggested that the networks were topologically closer to other observed TRNs than the *a priori* graphs $\mathcal {G}_{\mathcal {A}}$ and $\mathcal {G}_{\mathcal {AV}}$. Indeed, the original average degree (number of interactions per operon) of $\mathcal {G}_{\mathcal {A}}$ was 10.7, which is much higher than the values of 1.5–2.0 suggested in the literature [18] for a TRN. The resulting average degree of LOMBARDE outputs was 2.1, which is much closer to the expected value. This value is also close to the average degree value of 2.0 for the existing network $\mathcal {G}_{\mathcal {V}}$ of validated regulations for *E.coli*. Furthermore, the degree distribution (proportion of operons for each degree) in LOMBARDE outputs was highly correlated with the degree distribution in the existing network of validated regulations, which indicates that they shared some structural properties, as shown in Fig. 5. In contrast, the degree distributions in $\mathcal {G}_{\mathcal {A}}$ and $\mathcal {G}_{\mathcal {AV}}$ were significantly different; therefore, their structures were different from the structure of the observed network of validated regulations.

### Ranking of global regulators

The networks produced by LOMBARDE contained most of the global regulators that have been described for *E.coli* [19]. Starting from $\mathcal {G}_{\mathcal {A}}$, the output of LOMBARDE included 16 of the 19 known global regulators for this bacteria.

To determine the vertices that correspond to global regulators in the LOMBARDE output, we ranked them based on the connection structure of the network. In particular, we considered the vertex *radiality* to be a centrality index that measured the capability of each vertex to reach other ones in the graph [20]. If *d*(*u*,*v*) represents the distance from *u* to *v* (unweighted length of the shortest path from *u* to *v*) and *D* is the *diameter* of the graph ($D=\max \{d(\bar u,\bar v): \bar u, \bar v$ in the graph }), then the value *R*_*u*,*v*_=1+*D*−*d*(*u*,*v*) is minimal (with value 1) when *u* to *v* are the extreme vertices of the graph and maximal when vertices *u* and *v* are neighbors. Then the radiality *Rad*(*u*) of a vertex *u* is defined as the average of the values of *R*_*u*,*v*_ in LOMBARDE’s output. A vertex with high radiality is able to reach more vertices in fewer steps on average than a vertex with lower radiality.

We defined a vertex in LOMBARDE output to be a *central regulator* if its radiality index is ranked among the top 30 %. Ten of the known global regulators were among the central regulators in the LOMBARDE output (Table 2). In contrast, $\mathcal {G}_{\mathcal {A}}$ had only seven global regulators among the central regulators.

**Table 2:** *E. coli* global regulators and their ranking using radiality centrality index in the LOMBARDE output

Gene	Ranking	Ranking for radiality	Ranking for radiality
name	in literature	index in Lombarde	index in Lombarde
		output for $\mathcal {G}_{\mathcal {A}}$	output for $\mathcal {G}_{\mathcal {A}\mathcal {V}}$
crp	1	**25**	**1**
ihfA	2	**14**	**4**
ihfB	3	**16**	**5**
fnr	4	**1**	**6**
fis	5	63	**2**
arcA	6	**13**	**7**
lrp	7	**34**	87
hns	8	—	**14**
narL	9	121	126
ompR	10	143	96
fur	11	**7**	**8**
phoB	12	**9**	**25**
cpxR	13	80	**22**
soxR	14	69	**49**
soxS	15	109	**18**
mtfA	16	—	—
cspA	17	—	**42**
rob	18	**30**	95
purR	19	**39**	**47**

The first two columns list *E. coli* gene names and their global rankings as described in the literature. The last two columns show the ranking of the operons that contain each of these genes using the radiality index in the networks obtained by applying Lombarde to $\mathcal {G}_{\mathcal {A}}$ and $\mathcal {G}_{\mathcal {AV}}$. The numbers in bold indicate genes that were ranked among the top 30 % of radiality index and were considered central regulators

When LOMBARDE was applied to $\mathcal {G}_{\mathcal {AV}}$ (which also has the validated regulations in $\mathcal {V}$), 18 of the known global regulators were recovered in the output, 14 of them were central regulators. Therefore, LOMBARDE produced networks that gave a central role to most of the global regulators described in the literature.

### LOMBARDE applied to a meaningful set of co-expressions

**Fig. 6:**
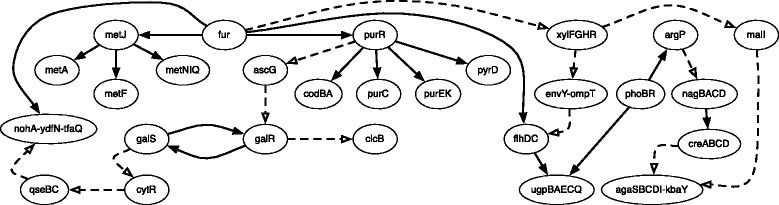
TRN obtained for a reduced set of co-regulated genes $\mathcal {C}'$ and the *a priori* graph $\mathcal {G}_{\mathcal {A}}$. All the arcs were predicted by LOMBARDE. The arcs drawn in solid lines have been validated experimentally, although this information was not used in the prediction. The gene *purC* was co-expressed with *metF*, *metA*, and *metQ*; *metN* was co-expressed with *purE* and *pyrD*; *metQ* was co-expressed with *codA*; *clcB* was co-expressed with *ydfN*; and *agaS* was co-expressed with *ugpA*

When the set of co-expressions $\mathcal {C}$ involved most of the genes in the organism, LOMBARDE produced a genome-wide putative TRN. However, LOMBARDE also can be applied to any specific set of biologically meaningful genes to decipher the regulatory relationships between them. As an illustration of this in the *ab initio* scenario, we restricted the set $\mathcal {C}$ obtained for *E.coli* to a subset $\mathcal {C}^{\prime }$ built of eight pairs of operons that contained co-expressed genes. From the *a priori* graph $\mathcal {G}_{\mathcal {A}}$, LOMBARDE produced the small putative regulatory network shown in Fig. 6. This network explained all the co-expressions using 30 regulations. In most cases, there was only one optimal explanation. For example, the gene *fur* codes for a transcription factor that regulates operons that contain *metA, metF, metNIQ, pyrD, purEK, purC*, and *codBA* through a regulatory cascade involving *metJ* and *purC*. In the other cases, there were several optimal explanations. For example, the co-expression of *nohA-ydfN-tfaQ* and *clcB* could be explained by cascades either from *fur* or from *galS*. Among the 30 regulations determined by LOMBARDE, 16 have been experimentally validated.

### Robustness of results for different input data

Input data used to generate the *a priori* graph used by LOMBARDE can vary according to the bioinformatics methods used to obtain them. By analyzing different ways of obtaining input data we found that LOMBARDE was quite robust to many variations. We also checked that, when the input data incorporated more information, as expected, LOMBARDE produced better predictions.

**Fig. 7:**
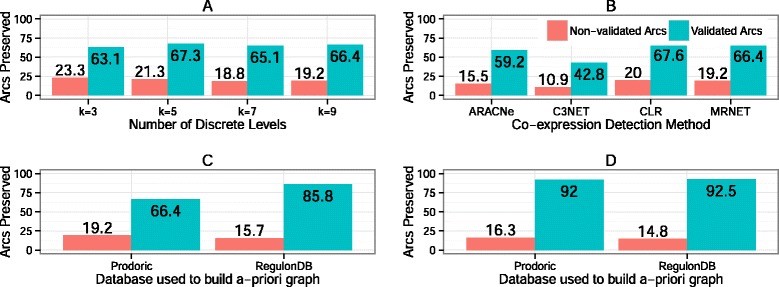
Comparisons of LOMBARDE performance with different input data. Different methods and parameters were used to generate the input *E. coli* data used by LOMBARDE. The percentage of arcs kept from the *a priori* graph in the output is shown in relation to the validated arcs in $\mathcal {V}$ and the non-validated arcs in $\mathcal {A}$. **a** The costs of the arcs in the *a priori* graph $\mathcal {G}_{\mathcal {A}}$ were computed for four values of the parameter *k*. **b** The set $\mathcal {C}$ was computed using four different methods to infer mutual information. **c** and **d** Two different databases were used to infer the set $\mathcal {A}$ and thus the *a priori* graphs $\mathcal {G}_{\mathcal {A}}$ and $\mathcal {G}_{\mathcal {AV}}$, respectively, were generated

For instance, the *a priori* graph depends on the source of the TF and BS patterns used to determine the set of TF/BS affinities. We compared the results of LOMBARDE applied to an *a priori* graph built using RegulonDB instead of the Prodoric database and found that LOMBARDE produced smaller graphs containing a greater proportion of validated arcs (Fig. 7, bar graph C). This is not surprising because Prodoric is based on several prokaryote organisms, while RegulonDB is based only on *E.coli*. However, the differences were not significant when validated regulations were included (Fig. 7, bar graph D).

The LOMBARDE results also were robust to the methods used to determine co-expressions. We evaluated LOMBARDE using different sets of co-expressions as determined by ARACNe [2], C3NET, CLR [3], and MRNET [4]. The LOMBARDE results were similar for two of the methods. The exception was C3NET (see bar graph B in Fig. 7), which produced the smallest set of co-expressions so LOMBARDE produced fewer explanations.

### Analysis of cost parameters

The cost of each arc in the *a priori* graph was chosen following two criteria: similar values are considered equivalent so that LOMBARDE results are robust to minor variations in *p*-value estimations; and cost should decrease as the arc *p*-value decreases so that arcs with higher likelihood have lower cost. The first criterion was fulfilled by classifying each arc into one of *k* bins and assigning a discrete cost based on the second criterion.

Without further restrictions the minimization algorithm will have to distinguish between alternative paths that have biological relevance: the regulatory cascade between two genes may be a short path with low confidence arcs or, alternatively, a long path with high confidence arcs. LOMBARDE will choose the longer path with high confidence arcs only when the path length is up to *r* times the length of the path with the low confidence arcs. Therefore the cost of an arc which belongs to the *i*-th bin (*i*∈{0,…,*k*−1}) is *r*^*i*^.

To choose useful values of *k* and *r* we explored the conditions that are summarized in Table 3. The size of the LOMBARDE output decreased when either *r* or *k* increased, and also when the number of validated arcs increased. The ratio of validated arcs among all the arcs in the LOMBARDE output increased with increased *k* and *r*. This result suggested that choosing the parameters *r*=10 and *k*=9 would bias LOMBARDE to produce more confident networks.

**Table 3:** Effects of parameters *k* and *r* on LOMBARDE output

	No. of arcs				No. of valid arcs				Ratio			
*r* ∖ *k*	3	5	7	9	3	5	7	9	3	5	7	9
1	24,471	24,471	24,471	24,471	428	428	428	428	1.8	1.8	1.8	1.8
1.2	15,184	14,339	13,093	12,051	385	375	363	369	2.5	2.6	2.8	3.1
1.5	12,204	10,299	8,951	8,059	369	344	338	333	3.0	3.3	3.8	4.1
2	10,294	8,423	7,144	6,453	351	338	325	314	3.4	4.0	4.5	4.9
5	6,379	5,612	4,999	4,955	293	300	294	295	4.6	5.3	5.9	6.0
10	5,958	5,466	4,817	4,922	280	299	289	295	4.7	5.5	6.0	6.0

The total number of arcs and the number of validated arcs in the Lombarde output decrease when *k* or *r* increase. When the ratio between these two numbers increased, more confident results were obtained; therefore, *k*=9 and *r*=10 gave the most confident results

## Conclusions

Deciphering which regulatory interactions can provide a causal explanation for a set of observed co-expressions remains an important challenge in systems biology. We developed LOMBARDE, a modeling method that uses an optimization principle to determine a simple and confident set of regulations that serve as causal explanations for a given set of co-expressions. When the set of co-expressions involves genome-wide interactions, LOMBARDE produces an explanatory putative TRN that has some basic topological characteristics close to observed TRNs and is biased to include regulations that have been independently validated experimentally. The LOMBARDE method was illustrated for a *E.coli* data set, where co-expressions were determined using mutual information under several environmental conditions. LOMBARDE was applied to an *a priori* graph considering TF/BS affinities recovered by BLAST and MEME/FIMO, and produced a simple and confident explanatory putative TRN that explained most of the observed co-expressions. LOMBARDE discarded a lot of arcs from the initial TRN but interestingly kept most of the independent experimentally validated regulations within it.

Sensitivity analysis showed that LOMBARDE was biased towards validated regulations in all cases of co-expression sets and *a priori* graphs used as input. Moreover, this method produced better results when the *a priori* graph was fine-tuned to the target organism.

We have evaluated LOMBARDE using *E.coli* as a test case because many regulatory interactions have been validated, making it is suitable for evaluation purposes. LOMBARDE can be applied in a straightforward way to other prokaryote organisms and we expect that the bias towards true regulations will be the same as long as the confidences of the predicted regulations are tuned to the target organism.

In summary, LOMBARDE is a tool that can provide useful insight into the regulatory mechanisms that underlie the phenotypical response of an organism to environmental challenges and it can be used as a reliable tool for further research on genome-scale transcriptional regulation studies.

## Availability of supporting data

Source code and raw data used for the evaluation are available at http://github.com/anaraven/Lombarde and as Additional file 1. The method can also be run from http://mobyle.inria.cl.

## Additional file

Additional file 1
**Source code and raw data used for the evaluation are available as Additional file 1.** (ZIP 1340 kb)
